# Blood Lead Exposure and Association With Hepatitis B Core Antibody in the United States: NHANES 2011–2018

**DOI:** 10.3389/fpubh.2022.873741

**Published:** 2022-06-14

**Authors:** Kexing Han, Tengyao He, Siran Huang, Weijie Sun, Yufeng Gao

**Affiliations:** Department of Infectious Diseases, The First Affiliated Hospital of Anhui Medical University, Hefei, China

**Keywords:** metals, serum lead, hepatitis B core antibody, NHANES, cross sectional survey

## Abstract

The objective of this project was to explore the distribution and related factors of blood lead and the association between blood lead and hepatitis B core antibody (HBcAb). All the data were from the U.S. National Health and Nutrition Examination Survey (NHANES). In total, 15,097 (aged 20–80 years) participants were included. Participants without a history of blood transfusion were more likely to be exposed to lower levels of blood lead [−2.30 (−3.13, −1.47) for HBcAb (–) and −2.23 (−4.54, 0.08) for HBcAb (+)]. The odds ratio (*OR*) of HBcAb (+) increased with blood lead and the result was 1.09 (1.06, 1.12). This study showed that older adults, men, people with a lower education level, a lower ratio of family income to poverty (PIR), a lower body mass index (BMI), or a history of blood transfusion, people who lived with a companion or with a total number of people in the family >3, people living in the United States for a longer time, U.S. citizens by birth or naturalization, and people not born in the United States were associated with higher blood lead exposure, and blood lead had a positive association with HBcAb (+).

## Introduction

Lead is a hazardous metal that is harmful to public health (1). Previous studies have confirmed that environmental lead exposure is associated with diseases of many systems, such as the nervous system, hematological system, and cardiovascular system (2–4). There is no safe minimum threshold for lead exposure (5). Lead occurs in small amounts in the earth's crust, but large amounts can be released into the natural environment by human activities, such as the burning of fossil fuels and the mining and chemical industry (6). Therefore, it is difficult to explore the factors of human exposure to lead in the natural environment. A prospective cohort study is the most effective research method to find the etiology, but this method is expensive, has high sensitivity to sample loss, and requires a lot of time to obtain effective data (7). Therefore, a regression cohort study has become an important method to study the factors related to lead exposure. Lead can be present in the body for a long time and this characteristic is the main reason why lead causes disease in patients. The relevant columns within the National Health and Nutrition Examination Survey (NHANES) database are kept up to date every 2 years and are commonly used for cross-sectional cohort studies. Studies on the distribution trends of blood lead in the U.S. population from the NHANES had been published since early times (8, 9). In recent years, many studies have focused on blood lead exposure in specific populations (10–12). Due to the uncontrollable nature of environmental factors, a growing number of studies have focused on factors associated with lead exposure on the participants' demographic characteristics, such as age, body mass index (BMI), and annual household income (13–15). However, to the best of our knowledge, there were few studies based on lead exposure in people with hepatitis B virus (HBV) infection.

The most sensitive index of HBV infection history, hepatitis B core antibody (HBcAB), indicates that the clinical states of patients are naturally diversified (16). However, it is worth noting that patients who are purely serum HBcAb positive remain a large group (17), and this kind of patient still has a probability of HBV reactivation, virologic breakthrough, virologic relapse, and becoming the communicator (18). The relationship between heavy metals exposure and viral hepatitis is becoming increasingly understood (19, 20), and viral hepatitis B is of greater concern because of its higher morbidity. Lead has been of interest to researchers as a representative heavy metal, and its exposure is insidious. Many diseases have been linked to the slow metabolism of lead in the body and its interference with immune regulation (21, 22). Lead toxicity and apoptosis in human cells involve a number of molecular processes, such as inducing cell death and oxidative stress (23) that also affect the hepatocytes infected with HBV (24). The long-term accumulation of heavy metals may eventually lead to the development of disease, and the search for causative factors related to HBcAb is of crucial importance in preventing the disease before it occurs. In addition to the known routes, sexual behavior, AIDS, and hepatitis C infection have all been linked to HBV infection (25), but the association between blood lead and HBcAb remains unknown.

In summary, this study analyzed the characteristics of blood lead distribution according to whether participants were seropositive for HBcAb or not in subgroups and explored the association between blood lead and HBcAb.

## Materials and Methods

### Study Participants

The NHANES is a study designed to assess the health and nutrition status of adults and children in the United States. The data in NHANES are updated every 2 years as a cycle. We collected four consecutive 2-year cycles (2011–2018) in the study, as these cycles contain the most complete information on blood lead and HBcAb. We included all participants aged 20–80 years who contained information on blood lead and HBcAb.

### Hepatitis B Core Antibody

The data of serology HBcAb were collected from the laboratory examination items in NHANES. This item contained information related to Serology HBcAb, HBV surface antigen, and hepatitis D antibody, and extracted the data we wanted to obtain and merged the data of four consecutive cycles. Participants with missing serological HBcAb information were removed.

### Blood Lead

In addition, blood lead data were obtained in the laboratory examination items from NHANES. Detailed information on laboratory quality assurance and monitoring is available at https://www.cdc.gov/nchs/nhanes/index.htm. Blood lead data from 2011 to 2018 were merged with the data of HBcAb by respondent sequence number.

### Covariates

Based on previous studies (12, 26), the following variables were included as covariates of serum lead: age, gender, race, education level, income to poverty (PIR), country of birth, citizenship status, length of stay in the United States, companion situation, and total number people in the family. The above covariates were derived from demographic variables and imputed with sample weights. The BMI of participants was collected from the examination data. In the projection of questionnaire data, the response to the questions “Ever told you have Hepatitis B?,” “Ever prescribed meds treat Hepatitis B?,” and “Ever receive blood transfusion?” were captured as the covariates for HBcAb. The data of covariates were combined according to the respondent sequence number of HBcAb.

### Statistical Analysis

We performed all statistical analyses by using R (http://www.R-project.org) and EmpowerStats (http://www.empowerstats.com), and the statistical significance was set at *p* < 0.05. In the calculation for all estimates, we used 2-year sample weights following the analytical guideline edited by the National Center for Health Statistics (NCHS) to promise the data of NHANES could represent the civilian non-institutionalized U.S. population. The classified variables were expressed in percentage (%) and the value of *p* was calculated by the weighted chi-square test. Continuous variables were denoted by means ± standard deviation (SD) and the value of *p* was calculated by a weighted linear regression model. If any covariate information was missing, the classified data were grouped separately, and the continuous data were processed using the average replacement. We showed the standardized regression coefficients (β), odds ratios (*OR*s), and 95% confidence intervals (95% *CI*s) in regression models. A weighted generalized additive model and a smooth curve fitting were conducted to address a non-linearity relationship, and we found cut-off points for segmentation effects. At the same time, subgroup analysis was used to find specific populations.

## Results

### Characteristics of the Participants

A total of 15,097 participants were included in our study, of whom 1,323 (8.76%) had HBcAb (+). Grouping the data by HBcAb, we knew that there were statistical differences in all characteristics between the two groups except “total number of people in the family.” Based on the analysis results, participants with HBcAb (+) would have a higher blood lead, older age, lower BMI, and a lower PIR than participants with HBcAb (–) (*p* < 0.01). Unexpectedly, participants who lived in the United States for more than 15 years and had a higher education level would tend to have a higher ratio of HBcAb (+) (*p* < 0.01). We show the demographic and laboratory data of the participants in Table 1.

**Table 1:** Characteristics of the U.S. participants in national health and nutrition examination survey (NHANES) from 2011 to 2018.

**Characteristics**	**HBcAb(+)** **(*n* = 1,323)**	**HBcAb(-)** **(*n* = 13,774)**	***P*-value**
Age (years)	55.4 ± 14.4	47.4 ± 17.0	<0.0001
Blood lead (ug/mL)	1.7 ± 1.5	1.2 ± 1.5	<0.0001
PIR	2.5 ± 1.5	3.0 ± 1.6	<0.0001
Body mass index (kg/m^2^)	28.5 ± 6.4	29.4 ± 7.0	0.0014
**Gender (%)**			<0.0001
Male	56.5	47.8	
Female	43.5	52.2	
**Race (%)**			<0.0001
Mexican American	3.4	8.8	
Other Hispanic	8.0	6.4	
Non-Hispanic White	36.8	66.7	
Other Race - Including Multi-Racial	24.2	10.2	
Unclear	27.6	7.9	
**Country of birth (%)**			<0.0001
Born in 50 US states or Washington, DC	54.7	83.1	
Others	45.3	16.9	
**Citizenship status (%)**			<0.0001
Citizen by birth or naturalization	81.7	91.1	
Others	18.3	8.9	
**Length of in US (%)**			<0.0001
1–5 years	5.3	2.1	
5–15 years	10.2	4.1	
≥15 years	28.8	9.8	
Unclear	55.8	84.0	
**Education level (%)**			<0.0001
Under high school graduate	23.3	13.4	
High school graduate	23.5	22.9	
More than high school graduate	53.1	63.6	
Unclear	0.1	0.0	
**Companion situation (%)**			0.0009
Living with companion	59.5	63.0	
Living alone	40.3	37.0	
Unclear	0.2	0.0	
**Total number people in the family (%)**			0.8604
≤3	68.3	68.0	
>3	31.7	32.0	
**Ever told you have hepatitis B (%)**			<0.0001
Yes	8.2	0.4	
No	61.0	67.0	
Unclear	30.8	32.6	
**Ever prescribed meds treat hepatitis B (%)**			<0.0001
Yes	1.8	0.1	
No	5.7	0.3	
Unclear	92.5	99.7	
**Ever receive blood transfusion (%)**			<0.0001
Yes	15.8	10.2	
No	83.4	88.6	
Unclear	0.8	1.2	

*Mean + SD for continuous variables: The value of p was calculated by weighted linear regression model*.

*Percentage for categorical variables: The value of p as calculated by the weighted chi-square test*.

### Factors Associated With Blood Lead

In this part, we showed standardized regression coefficients and 95% 95% *CI*s for three kinds of models grouped by HBcAb. The adjusted variables in each model were noted. As the classification indicators in all adjusted model, the risk factors associated with blood lead levels were men, other race (including multi-racial), missing information about ever being informed to have HBV, did not prescribe medicines to treat HBV, had a history of blood transfusion, born in other countries, citizen by birth or naturalization, unclear for the length of stay in the United States, under high school graduate, living with companion, and total number of people in the family ≤3 for HBcAb (+) participants, and male, other race (including multi-racial), missing information about the HBV status, prescribed medicines to treat HBV, had a history of blood transfusion, born in all 50 states or Washington, D.C, citizenship in others, more than 15 years of stay in the United States, under high school graduate, living with a companion, and total number of people in the family ≤3 for HBcAb (–) participants. The blood lead tended to increase with the increase of age in three kinds of models, and we did the trend test for the phenomenon (*p* for trend < 0.01). In the analysis of the association between BMI with blood lead, we found participants who had higher BMI would have lower serum lead in all the models, and *p* for trend < 0.01. This trend was more obvious in HBcAb (+) participants. The standardized regression coefficient and 95% *CI* were −6.24 (95% *CI*: −13.29, 0.81) for BMI >23.9 when we adjusted “age, gender, and race.” Participants who had lower PIR would be harmed from higher blood lead in the non-adjusted model and adjusted “age, gender, and race” model, and *p* for trend < 0.05. Although there was no statistical difference for the trend test among HBcAb (+) in the all-adjusted model, the regression coefficient (−1.30) suggested that those participants with higher PIR had a lower blood lead potentially (*p* for trend = 0.55). Table 2 displays the above.

**Table 2:** Associations between hypothesized risk factors and blood lead levels (ug/L).

**Characteristics**	**Model 1**		**Model 2**		**Model 3**	
	**HBcAb (–)** **β(95%CI)**	**HBcAb (+)** **β(95%CI)**	**HBcAb (–)** **β(95%CI)**	**HBcAb (+)** **β(95%CI)**	**HBcAb (–)** **β(95%CI)**	**HBcAb (+)** **β(95%CI)**
**Gender**						
Male	Ref.	Ref.	Ref.	Ref.	Ref.	Ref.
Female	−4.29(−4.80, −3.79)	−4.16(−5.81, −2.51)	−4.53(−5.02, −4.04)	−4.74(−6.36, −3.13)	−4.18(−4.68, −3.69)	−4.52(−6.21, −2.84)
**Race**						
Mexican American	Ref.	Ref.	Ref.	Ref.	Ref.	Ref.
Other Hispanic	−3.51(−4.82, −2.20)	1.44(−3.89, 6.77)	−3.59(−4.85, −2.32)	1.90(−3.26, 7.06)	−2.94(−4.26, −1.63)	2.90(−2.46, 8.26)
Non–Hispanic White	−0.96(−1.87, −0.06)	3.20(−1.47, 7.87)	−2.53(−3.41, −1.64)	2.80(−1.71, 7.32)	1.89(0.84, 2.94)	3.06(−1.77, 7.89)
Other Race – Including Multi–Racial	−0.47(−1.63, 0.69)	6.30(1.53, 11.07)	−0.80(−1.92, 0.32)	7.26(2.64, 11.88)	2.36(1.11, 3.60)	6.59(1.77, 11.42)
Unclear	−0.31(−1.55, 0.93)	4.44(−0.29, 9.17)	−0.73(−1.93, 0.47)	5.12(0.54, 9.70)	1.72(0.43, 3.01)	6.58(1.74, 11.42)
**Age (years)**						
20–39	Ref.	Ref.	Ref.	Ref.	Ref.	Ref.
40–59	4.34(3.75, 4.92)	5.76(3.41, 8.12)	4.48(3.91,5.06)	5.82(3.49, 8.15)	4.77(4.18, 5.36)	6.82(4.36, 9.27)
60–80	7.31(6.68, 7.94)	9.42(7.02, 11.82)	7.76(7.13,8.40)	9.80(7.43, 12.18)	7.47(6.79, 8.16)	10.04(7.44, 12.63)
**P for trend <0.01**						
**BMI (kg/m** ^**2**^ **)**						
<18.5	Ref.	Ref.	Ref.	Ref.	Ref.	Ref.
18.5–23.9	−0.46(−2.60, 1.68)	−2.87(−10.30, 4.55)	−1.63(−3.69, 0.43)	−2.89(−10.04, 4.26)	−0.13(−2.24, 1.98)	−0.17(−2.19, 1.85)
>23.9	−1.40(−3.48, 0.68)	−5.48(−12.76, 1.80)	−4.06(−6.08, −2.05)	−6.24(−13.29, 0.81)	−1.29(−3.34, 0.76)	−1.40(−3.37, 0.57)
***P*** **for trend <0.01**
**PIR**						
<1.30	Ref.	Ref.	Ref.	Ref.	Ref.	Ref.
1.30–3.50	−1.17(−1.86, −0.48)	−0.92 (−2.93, 1.09)	−2.26 (−2.94, −1.58)	−1.07(−3.02, 0.88)	−0.31(−1.01, 0.39)	−0.02(−2.07, 2.03)
≥3.50	−2.33(−3.02, −1.63)	−2.86(−5.11, −0.61)	−3.68(−4.37, −2.98)	−2.62(−4.88, −0.36)	−0.83(−1.59, −0.07)	−1.30(−3.77, 1.17)
***P*** **for trend <0.05**						***P*** **for trend** **=** **0.55**
**Ever told you have hepatitis B**						
Yes	Ref.	Ref.	Ref.	Ref.	Ref.	Ref.
No	−0.17(−4.19, 3.85)	0.17(−2.90, 3.24)	2.10 (−1.78, 5.98)	−0.54(−3.53, 2.45)	2.74(−8.87, 14.35)	0.13(−9.42, 9.67)
Missing	2.96 (−1.07, 6.99)	0.33(−2.92, 3.57)	5.38(1.49, 9.28)	0.27(−2.90, 3.44)	5.60(−6.02, 17.21)	0.51(−9.07, 10.10)
**Ever prescribed meds treat hepatitis B**						
Yes	Ref.	Ref.	Ref.	Ref.	Ref.	Ref.
No	−0.01(−9.72, 9.69)	4.33(−2.80, 11.47)	−2.12(−11.49, 7.25)	4.08(−2.85, 11.01)	−0.46(−9.98, 9.05)	4.00(−3.12, 11.12)
Missing	0.50(−7.82, 8.83)	3.52(−2.78, 9.81)	1.71(−6.33, 9.75)	3.12(−2.99, 9.23)	−3.79(−17.98,10.39)	3.61(−7.73, 14.95)
**Ever receive blood transfusion**						
Yes	Ref.	Ref.	Ref.	Ref.	Ref.	Ref.
No	−2.61(−3.45, −1.78)	−2.45(−4.71, −0.18)	−0.63(−1.46, 0.21)	−2.86(−5.13, −0.58)	−2.30(−3.13, −1.47)	−2.23(−4.54, 0.08)
Missing	−1.39(−3.87, 1.08)	−1.49(−10.74, 7.76)	−2.32(−4.71, 0.07)	−2.96(−11.89, 5.97)	−1.32(−3.75, 1.12)	−1.90(−11.06, 7.27)
	**HBcAb (–)** **β(95%CI)**	**HBcAb (+)** **β(95%CI)**	**HBcAb (–)** **β(95%CI)**	**HBcAb (+)** **β(95%CI)**	**HBcAb (–)** **β(95%CI)**	**HBcAb (+)** **β(95%CI)**
**Country of birth**						
Born in 50 US states or Washington, DC	Ref.	Ref.	Ref.	Ref.	Ref.	Ref.
Others	2.19(1.51, 2.86)	−2.17(−3.83, −0.51)	3.39(2.58, 4.20)	−3.16 (−5.38, −0.94)	−2.20(−5.01, 0.61)	2.41(−5.87, 10.69)
**Citizenship status**						
Citizen by birth or naturalization	Ref.	Ref.	Ref.	Ref.	Ref.	Ref.
Others	3.68(2.79, 4.57)	−0.72(−2.86, 1.42)	5.48(4.51, 6.46)	1.22(−1.03, 3.47)	3.05 (1.67, 4.42)	−0.32(−3.18, 2.54)
**Length of in US (years)**						
1–5	Ref.	Ref.	Ref.	Ref.	Ref.	Ref.
5–15	1.37(−0.78, 3.51)	0.28(−4.15, 4.70)	0.49(−1.59, 2.57)	−1.65(−5.93, 2.63)	1.67(−0.47, 3.82)	0.23(−4.27, 4.73)
≥15	0.93(−1.00, 2.85)	−0.46(−4.37, 3.44)	−2.25(−4.13, −0.38)	−3.80(−7.67, 0.06)	2.68(0.61, 4.75)	−0.10(−4.49, 4.29)
Unclear	−1.31(−3.08, 0.46)	2.13(−1.63, 5.89)	−4.59(−6.35, −2.83)	0.57(−3.41, 4.55)	−0.26(−3.42, 2.90)	5.58(−3.33, 14.49)
**Education level**						
Under high school graduate	Ref.	Ref.	Ref.	Ref.	Ref.	Ref.
High school graduate	−2.84(−3.70, −1.97)	−2.59(−5.00, −0.19)	−2.56(−3.42, −1.71)	−2.38(−4.74, −0.02)	−2.09(−2.97, −1.20)	−2.78(−5.22, −0.34)
More than high school graduate	−5.38(−6.13, −4.62)	−4.41(−6.45, −2.37)	−4.75(−5.52, −3.97)	−4.05(−6.10, −2.00)	−4.85(−5.67, −4.03)	−4.04(−6.26, −1.81)
Unclear	−2.50(−15.12, 10.13)	−8.56(−31.94, 14.81)	−7.50(−19.72, 4.72)	−5.15(−27.75, 17.46)	−3.04(−15.75, 9.66)	−3.16(−28.39, 22.07)
**Companion situation**						
Living with Companion	Ref.	Ref.	Ref.	Ref.	Ref.	Ref.
Living alone	−0.02(−0.54, 0.51)	0.84(−0.85, 2.53)	1.01(0.50, 1.53)	0.13(−1.57, 1.83)	−0.73(−1.27, −0.19)	−0.44(−2.23, 1.35)
Unclear	−1.90(−24.50, 20.69)	−3.62(−22.51, 15.28)	−4.17(−25.97, 17.62)	−5.93(−24.18, 12.33)	−2.32(−24.84, 20.21)	−5.81(−26.10, 14.47)
**Total number people in the family**						
≤3	Ref.	Ref.	Ref.	Ref.	Ref.	Ref.
>3	−1.96(−2.50, −1.41)	−0.35(−2.13, 1.43)	−0.28(−0.84, 0.27)	1.46(−0.41, 3.32)	−2.63(−3.18, −2.07)	−0.32(−2.19, 1.56)

*Model 1: No covariates were adjusted*.

*Model 2: Age, gender, and race were adjusted*.

*Model 3: All the covariates were adjusted*.

^*^In the subgroup analysis stratified by each covariate, the model is not adjusted for the stratification variable itself.

**Figure 1:**
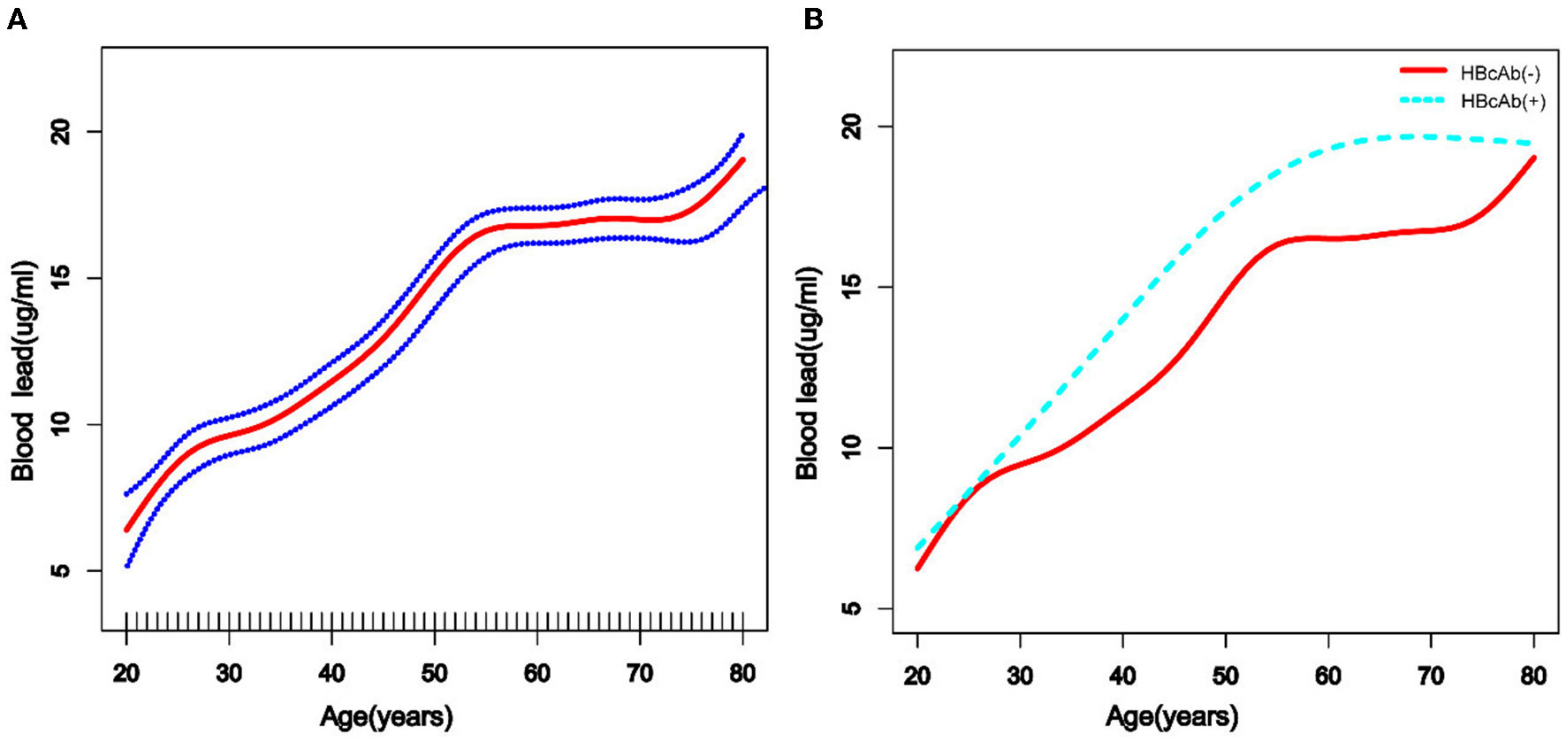
The association between age and blood lead levels. **(A)** The solid rad line represents the smooth curve fit between variables and a dotted line represents the 95% confidence interval (*CI*) from the fit. All the covariates in the study and Hepatitis B core antibody (HBcAb) were adjusted except age. **(B)** The solid rad line represents HBcAb (–) and the dotted line represents HBcAb (+). All the covariates in the study are adjusted.

**Figure 2:**
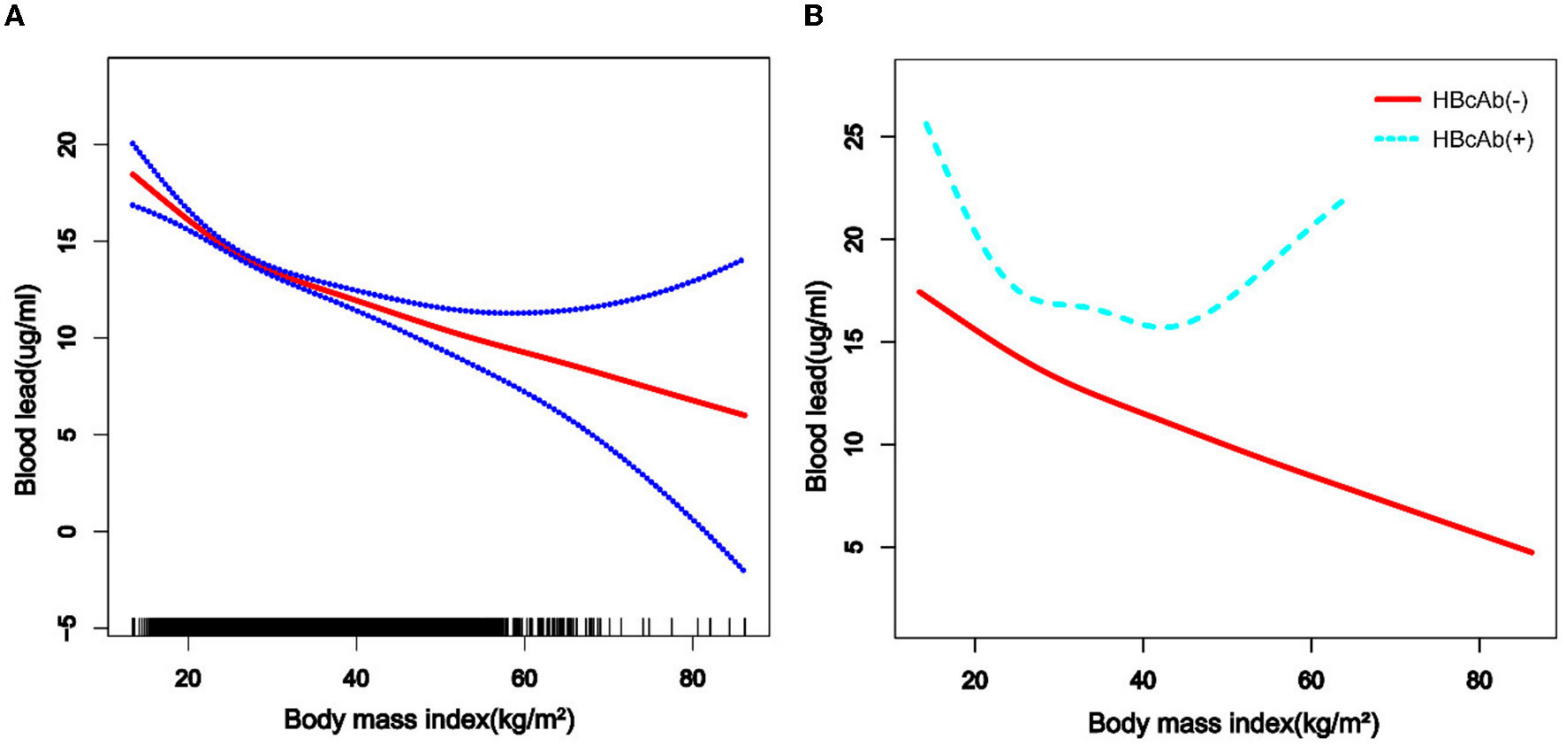
The association between BMI and blood lead levels. **(A)** The solid rad line represents the smooth curve fit between variables and a dotted line represents the 95% *CI* from the fit. All the covariates in the study and HBcAb were adjusted except body mass index (BMI). **(B)** The solid rad line represents HBcAb (–) and the dotted line represents HBcAb (+). All the covariates in the study are adjusted.

**Figure 3:**
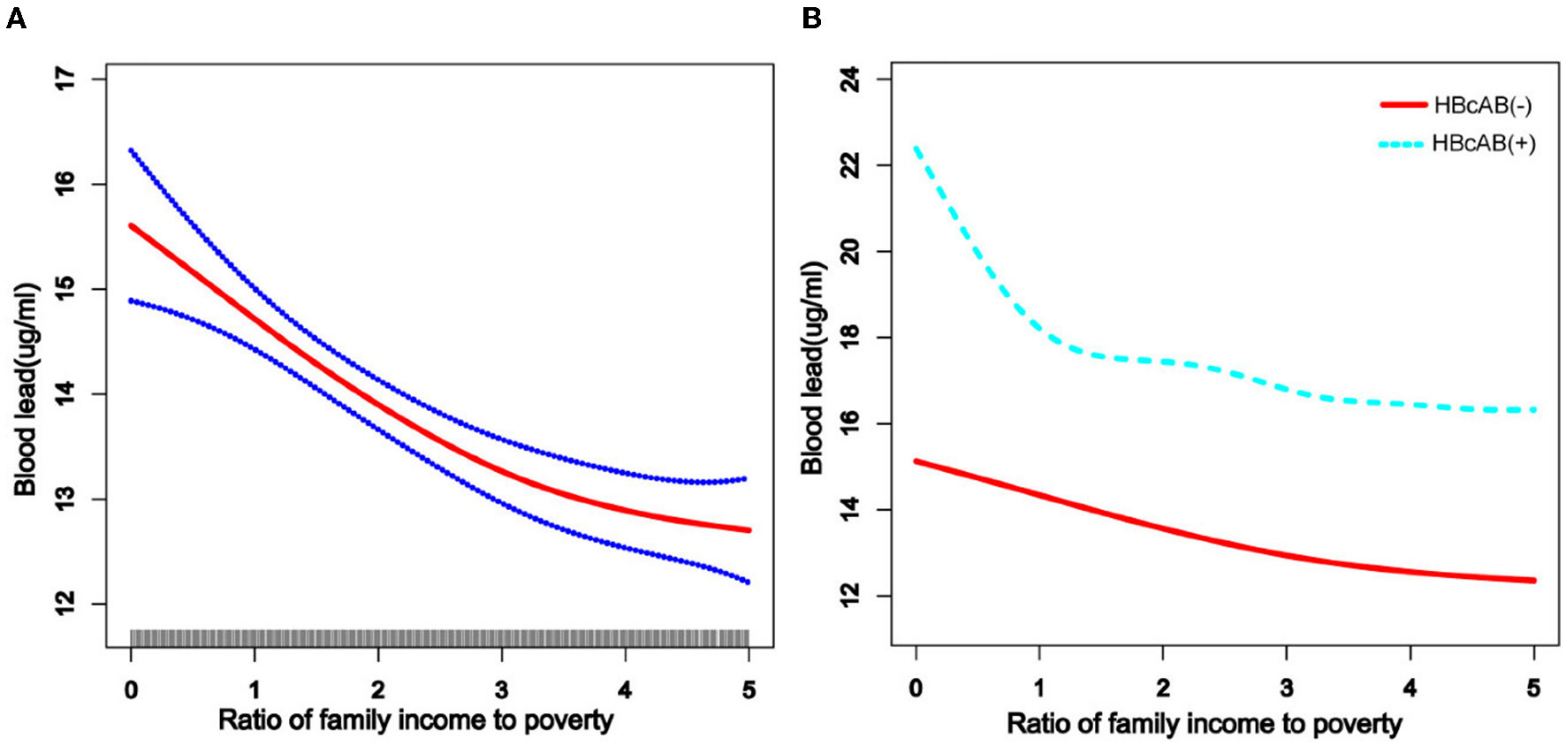
The association between income to poverty (PIR) and blood lead levels. **(A)** The solid rad line represents the smooth curve fit between variables and a dotted line represents the 95% *CI* from the fit. All the covariates in the study and HBcAb were adjusted except PIR. **(B)** The solid rad line represents HBcAb (–) and the dotted line represents HBcAb (+). All the covariates in the study are adjusted.

We used a weighted generalized additive model and a smooth curve fitting to address the non-linear relationship and select the corresponding model according to the result of the logarithmic likelihood ratio (LLR) for threshold effect analysis. The non-linear model was selected when the LLR <0.05. In the association between age and blood lead, we found a non-linear relationship and obvious cut-off points for all the participants, and the non-linear relationship and cut-off points also existed when the participants were grouped by HBcAb (Figure 1). Models and cut-off points are shown in Table 3. The non-linear relationship between BMI and blood lead for all participants and participants sorted by HBcAb is shown in Figure 2. The effective BMI cut-off points for HBcAb (+) participants were 24.8, 31.8, and 53.7. Models and cut-off points are shown in Table 4. Although there was a non-linear relationship between PIR and blood lead (Figure 3), the cut-off point for PIR was not statistically different (LLR >0.05), so we chose a linear model in this study (Table 5, Figure 3).

**Table 3:** Cut-off points and segmentation effects for age (years).

**Indexes**	**Cut-off points for overall**	Overall β (95% CI)^*^	**Cut-off points for HBcAb (-)**	HBcAb (-) β (95% CI)^**^	**Cut-off points for HBcAb (+)**	HBcAb (+) β (95% CI)^**^
**Model 1**	Non	0.20 (0.19, 0.22)	Non	0.20 (0.18, 0.22)	Non	0.22 (0.16, 0.28)
**Model 2**						
**Age (years)**						
Cut-off point 1	20–26	0.29 (0.07, 0.52)	20–26	0.30 (0.06, 0.53)	20–72	0.30 (0.23, 0.37)
Cut-off point 2	26–56	0.24 (0.21, 0.28)	26–56	0.24 (0.20, 0.27)	≥72	−0.60 (−1.01, −0.18)
Cut-off point 3	56–78	0.09 (0.04, 0.15)	56–79	0.09 (0.03, 0.14)		
Cut-off point 4	≥78	2.30 (-0.91, 5.50)	≥79	3.29 (1.95, 4.62)		

*Model 1: A straight line effect model; Model 2: Segmentation effects model by cut-off points*.

* *Gender, race, body mass index (BMI), income to poverty (PIR), hepatitis B core antibody (HBcAb), ever informed about hepatitis B status, ever prescribed medicines to treat hepatitis B, ever received blood transfusion, country of birth, citizenship status, length of stay in the United States, education level, companion situation, and total number of people in the family were adjusted*.

** *Gender, race, BMI, PIR, ever informed about hepatitis B status, ever prescribed medicines to treat hepatitis B, ever received blood transfusion, country of birth, citizenship status, length of stay in the United States, education level, companion situation, and total number of people in the family were adjusted*.

**Table 4:** Cut-off points and segmentation effects for BMI (kg/m^2^).

**Indexes**	**Cut-off points for overall**	Overall β (95% CI)^*^	**Cut-off points for HBcAb (-)**	HBcAb (-) β (95% CI)^**^	**Cut-off points for HBcAb (+)**	HBcAb (+) β (95% CI)^**^
**Model 1**	Non	−0.17(−0.20, −0.14)	Non	−0.17(−0.20, −0.13)	Non	−0.21(−0.35, −0.08)
**Model 2**						
**BMI (kg/m** ^**2**^ **)**						
Cut-off point 1	<26.1	−0.37(−0.50, −0.24)	<26.1	−0.36(−0.49, −0.22)	<24.8	−1.17(−1.80, −0.54)
Cut-off point 2	≥26.1	−0.12(−0.17, −0.08)	≥26.1	−0.12(−0.17, −0.08)	24.8–31.8	0.54(0.04, 1.03)
Cut-off point 3					31.8–53.7	−0.34(−0.66, −0.02)
Cut-off point 4					≥53.7	0.72(−0.74, 2.18)

*Model 1: A straight line effect model; Model 2: Segmentation effects model by cut-off points*.

* *Gender, race, age, PIR, HBcAb, ever informed about hepatitis B status, ever prescribed medicines to treat hepatitis B, ever received blood transfusion, country of birth, citizenship status, length of stay in the United States, education level, companion situation, total number of people in the family were adjusted*.

** *Gender, race, age, PIR, ever informed about hepatitis B status, ever prescribed medicines to treat hepatitis B, ever received blood transfusion, country of birth, citizenship status, length of stay in the United States, education level, companion situation, total number of people in the family were adjusted*.

**Table 5:** Cut-off points and segmentation effects for ratio of family PIR.

**Indexes**	**Cut-off points for overall**	Overall β (95% CI)^*^	**Cut-off points for HBcAb (-)**	HBcAb (-) β (95% CI)^**^	**Cut-off points for HBcAb (+)**	HBcAb (+) β (95% CI)^**^
**Model 1**	Non	−0.49(−0.65, −0.32)	Non	−0.48(−0.66, −0.31)	Non	−0.49(−1.08, 0.10)
**Model 2**						
**PIR**						
Cut-off point 1	<4.35	−0.63(−0.89, −0.38)	<4.35	−0.64(−0.90, −0.37)	<1.39	−1.53 (−4.33, 1.27)
Cut-off point 2	≥4.35	0.41(-0.76, 1.58)	≥4.35	0.45(−0.77, 1.67)	≥1.39	−0.32 (−1.06, 0.41)
Cut-off point 3						

*Model 1: A straight line effect model; Model 2: Segmentation effects model by cut-off points*.

* *Gender, race, age, BMI, HBcAb, ever informed about hepatitis B status, ever prescribed medicines to treat hepatitis B, ever received blood transfusion, country of birth, citizenship status, length of stay in the United States, education level, companion situation, total number of people in the family were adjusted*.

** *Gender, race, age, PIR, ever informed about hepatitis B status, ever prescribed medicines to treat hepatitis B, ever received blood transfusion, country of birth, citizenship status, length of stay in the United States, education level, companion situation, total of number people in the family were adjusted*.

### Blood Lead and HBcAb

**Figure 4:**
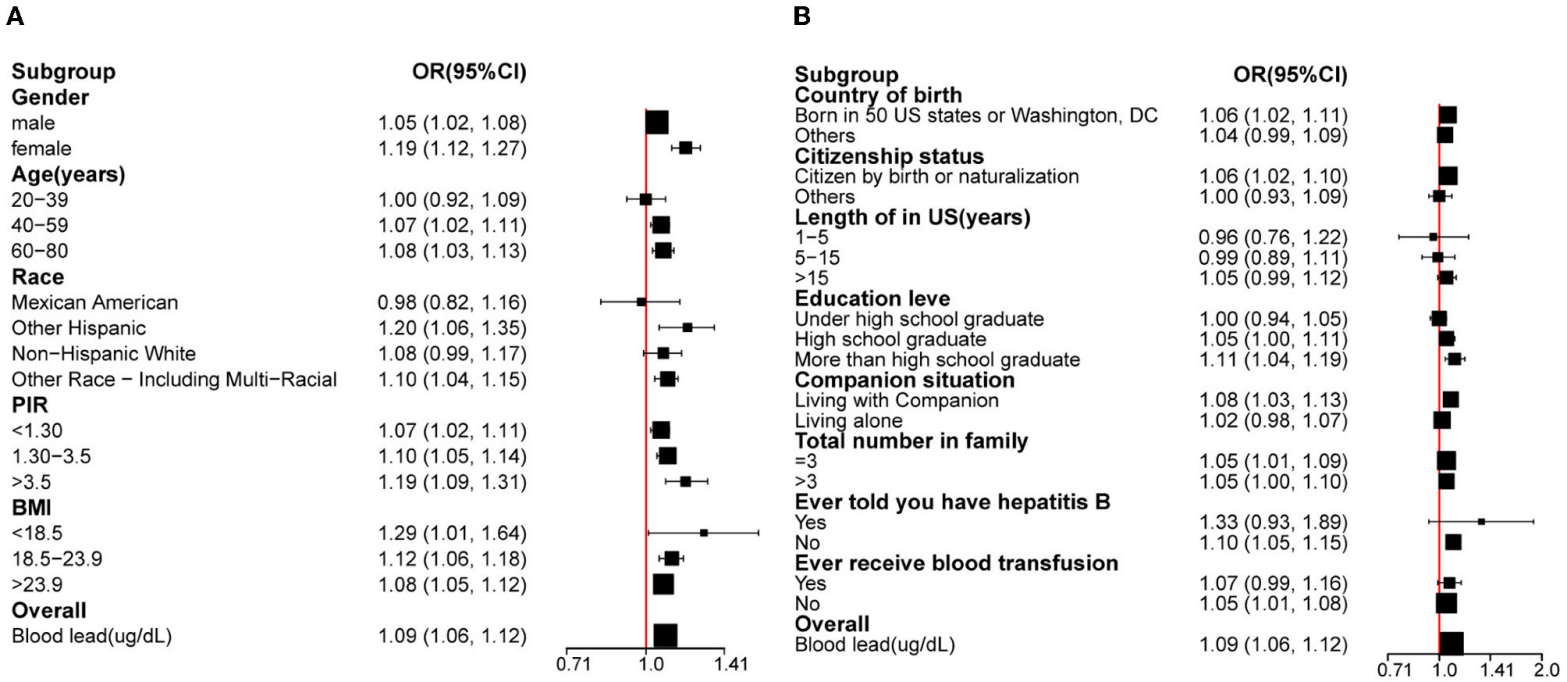
The association between blood lead levels and HBcAb. **(A)** Subgroup analysis was performed according to gender, race, age, and BMI. **(B)** Subgroup analysis was performed according to the country of birth, citizenship status, length of stay in the United States, education level, companion situation, total number of people in the family, ever informed about hepatitis B status, and ever received blood transfusion. *All the covariates are in the fully adjusted model. In the subgroup analysis stratified by each covariate, the model is not adjusted for the stratification variable itself.

In this part, we converted the unit of serological HBcAb into deciliters and multiplied all the data by 10 to explore the association between blood lead and HBcAb at a lower concentration. After multivariate adjustment for the above-mentioned covariates, an increased odds of HBcAb (+) was observed across the blood lead in all participants. The *OR* and 95% *CI* were 1.09 (95% *CI*: 1.06, 1.12). Additionally, we performed subgroup analysis based on population data. As blood lead levels increased, participants with the characteristics of female, age between 60 and 80 years, other Hispanic, PIR >3.5, BMI <18.5, born in 50 U.S. states or Washington, D.C, citizen by birth or naturalization, lived in the United States for more than 15 years, more than high school graduate, and living with a companion were more likely to be HBcAb (+). Results of subgroup analysis are shown in Figure 4. The *OR*s and 95% *CI*s of participants who received antiviral treatment and those who did not were 2.55 (95% *CI*: 0.54, 11.97) and 1.34 (95% *CI*: 0.91, 1.98), and this result was presented separately here because the 95% *CI*s were beyond the limit.

## Discussion

According to previous studies, the diversity of blood lead levels in different age groups was complex, with younger children and older adults tending to have higher blood lead levels (14, 15, 26). Similar results were obtained in the participants of this study, while trend tests showed that participants' blood lead levels increased with age and that this trend was more pronounced in participants with HBcAb (+). Lead exposure could increase the prevalence of metabolic disorders, and the risk of metabolic disorders increases with age (27). We believe that metabolic diseases might alter the mechanisms by which lead is metabolized in the body, which could also explain the higher blood lead levels in the older participants.

As depicted in previous studies, blood lead levels were higher in low-income, male, and non-Hispanic populations (9). At the same time, we found that born in other countries, citizen in other ways, longer stays in the United States, under high school graduate, living with a companion, and total number of people in the family ≤3 might be risk factors associated with blood lead level. Although there have been few studies to explain why people with these characteristics had higher blood lead levels, we know that lead exposure is occupational and is associated with poor living conditions (28, 29), so we speculate that the participants in this study with higher blood lead levels might have been at a higher risk of lead exposure due to their work and living conditions. For example, people with lower levels of education might be less aware of protection at work (30). Blood lead levels tended to be lower among participants with higher PIR, but the trend was not as smooth among participants with HBcAb (+) as it was among participants with HBcAb (–). However, it is undeniable that people with higher PIR have lower blood lead levels (31), and our study suggests that this negative relationship was more pronounced in participants with HBcAb (+) (although no statistical difference was observed in our study), which we believe could be inextricably linked to better health insurance.

Studies have shown that single heavy metal level in urine was negatively correlated with BMI (32), but the mixed dataset of heavy metal levels was positively correlated with body fat percentage (33). Our study suggested that serum lead levels tend to decrease with increasing BMI, and the cut-off point equal to 26.1 was important for all populations. The above tendency for blood lead levels would slow down when the BMI was >26.1. There was a U-shaped relationship between blood lead levels and HBcAb in participants with HBcAb (+), and participants might have had higher blood lead levels when their BMI exceeded 53.7 kg/m^2^, but such results still need to be confirmed by more studies. The mechanism of why participants with higher BMI had lower blood lead levels instead requires further explanation in subsequent studies.

A purely positive serological HBcAb is defined as having been infected with HBV, although the patient's disease status after HBV infection still needs to be combined with other indicators, but such patients still have the possibility of HBV reactivation (34). Reactivation of HBV is closely linked to changes in immunity, especially when the patient is immunosuppressed. Lead exposure could lower immunity and lead to many diseases. Previous studies had shown that lead exposure resulted in a lower positive rate of hepatitis B surface antibodies (Anti-HBs) in children (35), which suggested that lead exposure could change the body's immune response to HBV. Our study demonstrated a positive correlation between blood lead levels and HBcAb (+). Although mechanistic studies on the association between serum lead and HBcAb are still unreported, an alteration in the immune status of the body caused by lead exposure might be a potential mechanism to explain this positive relationship. In a subgroup analysis, we found that participants who were women, had higher levels of education, and had higher PIR were more likely to have higher *OR* with the increasing blood lead levels. The timely detection of HBV infection is related to many factors, one of which is the frequency of medical institution visits. Previous studies indicated that the management of HBV would increase a huge economic burden on both society and individuals (36, 37). People with more education had a tendency to have a higher awareness of disease (38) and adult women required comprehensive prenatal laboratory testing (39). These reasons might be a satisfactory explanation for the results of the subgroup analysis. In our study, participants with a higher BMI tended to have lower blood lead levels. However, the mechanism of the phenomenon had not been reported. Some studies have shown that the positive expression rate of hepatitis B surface antigen (HBsAg) and Anti HBs could decrease with the increase of BMI (40, 41). Whether the potential mechanisms of these two results are similar still needs to be confirmed by subsequent studies.

Cross-sectional studies inevitably have missing variables, and the presence of missing variables could bias the phenomenon of interest. In this study, there were no missing independent or respondent variables. We reduced this bias by setting up separate groups for missing covariates, and we built three adjustment models in the regression analysis to reduce the effect of confounding factors, so that the conclusions obtained from our study were reliable. In addition, some of the indicators included in this study were derived from questionnaires, so there was potential for recall bias, but we believe that NHANES items were designed with good quality control for such bias and therefore did not affect the final results. Finally, blood lead exposure in this study was based on demographic characteristics and we cannot guarantee that all information on participants could be included, so our results would need to be confirmed and enriched by additional studies. Finally, the biggest limitation of cross-sectional studies is that they cannot be used to explain causality, so more studies are needed to explore the causal link between blood lead level and HBcAb.

In this study, we first determined that demographic characteristics and blood lead levels differed between the HBcAb (+) and HBcAb (–) groups. Among participants with previous HBV infection, there was a higher likelihood of higher blood lead levels among men of lower economical means and lower levels of education compared with other participants, and this risk had the potential to increase with age. Finally, we confirmed a positive relationship between blood lead and positive expression of HBcAb.

## Data Availability Statement

Publicly available datasets were analyzed in this study. This data can be found here: https://wwwn.cdc.gov/nchs/nhanes/Default.aspx.

## Author Contributions

Material preparation, data collection, and analysis were performed by KH, TH, WS, and YG. The first draft of the manuscript was written by KH and all authors commented on previous versions of the manuscript. All authors contributed to the study conception and design, read, and approved the final manuscript.

## Conflict of Interest

The authors declare that the research was conducted in the absence of any commercial or financial relationships that could be construed as a potential conflict of interest.

## Publisher's Note

All claims expressed in this article are solely those of the authors and do not necessarily represent those of their affiliated organizations, or those of the publisher, the editors and the reviewers. Any product that may be evaluated in this article, or claim that may be made by its manufacturer, is not guaranteed or endorsed by the publisher.
